# Arthroscopic Viewing Position Affects Anterior Cruciate Ligament Reconstruction Femoral Tunnel Length Measurements

**DOI:** 10.3389/fsurg.2018.00016

**Published:** 2018-03-01

**Authors:** Sheeba M. Joseph, Michael R. Karns, Derrick M. Knapik, James E. Voos

**Affiliations:** ^1^Department of Orthopaedic Surgery, University Hospitals Cleveland Medical Center, Cleveland, OH, United States; ^2^Sports Medicine Institute, University Hospitals Cleveland Medical Center, Cleveland, OH, United States

**Keywords:** ACL reconstruction, arthroscopy, femoral tunnel length, seventy-degree arthroscopy, graft-tunnel mismatch

## Abstract

**Purpose:**

To purpose of this study was to compare arthroscopic anterior cruciate ligament (ACL) reconstruction femoral tunnel length measurements from the anterolateral portal between the standard notch view using a 30° arthroscope versus a “top-down” view utilizing a 70° arthroscope to visual the far side of the femoral tunnel aperture.

**Methods:**

Arthroscopic femoral tunnel length measurements using calibrated reamers from the standard notch versus the “top-down” view were obtained and reviewed in 54 skeletally mature patients undergoing ACL reconstruction with no prior bony knee surgery. Patient age, height, weight, sex, and surgery laterality were also recorded. Measurements of femoral tunnel length were repeated using both views for inter-observer and intra-observer correlation.

**Results:**

Inter-observer and intra-observer intra-class correlation coefficients for the standard notch view and “top-down” views were excellent, with higher reliability values appreciated using the “top down” view. Mean overall femoral tunnel length measurements obtained using the standard notch view were significantly longer than measurements from the “top-down” view (*p* < 0.001).

**Conclusions:**

The standard notch view provides significantly longer femoral tunnel length measurements in comparison to the “top-down” view.

## Introduction

As of 2015, over 200,000 anterior cruciate ligament (ACL) reconstructions are performed each year in the United States.(1–3) To improve patient outcomes and return to play rates, advances in surgical techniques have sought to achieve more anatomic reconstructions.(4) As such, multiple techniques have been developed to enhance the surgeon’s ability to create the femoral tunnel at the anatomic origin of the ACL including drilling from an accessory medial portal with the knee in a hyper-flexed (~120°) position.(5, 6) However, drilling the femoral tunnel at such a low, posterior position in the hyper-flexed knee using rigid reamers increases the risks for short tunnels, posterior cortical blowout, and common peroneal nerve injury if the guide pins exits inferior to the biceps femoris.(7–10) Several commercially available flexible reamer systems have been developed permitting drilling of an anatomically placed femoral tunnel closer to 90° of knee flexion, resulting in longer tunnel lengths and safer distances from the common peroneal nerve.(11, 12)

Achieving anatomic femoral tunnel placement and subsequent graft insertion demands adequate visualization of the intercondylar notch wall of the lateral femoral condyle. Osaki et al examined the femoral tunnel aperture within the intercondylar notch and demonstrated a discrepancies of up to 5 mm using an outside-in drilling technique and 4.2 mm with a trans-portal drilling technique between tunnel lengths measured at the center versus the shortest aspects of the femoral tunnel aperture.(13) The authors attributed their findings to the obliquity of the femoral tunnel relative to the lateral femoral intercondylar notch wall. Accordingly, the anatomy and inevitable oblique orientation for femoral tunnel drilling may result in overestimation of tunnel length if the near side (i.e., distal aspect) of the aperture is referenced versus referencing the far side (i.e., proximal aspect) of the aperture. Such tunnel length inaccuracy may lead to a proud plug with use of a bone-tendon-bone graft or an insufficient graft within the tunnel using soft-tissue only grafts.(14)

Visualizing the entire femoral ACL footprint can be challenging from a standard anterolateral-viewing portal using a 30° arthroscope. Several authors have described improved visualization of the femoral ACL footprint using a 70° arthroscope from the lateral portal.(14, 15) Moreover, use of the 70° arthroscope using the standard anterolateral viewing portal enables the surgeons to achieve a “top-down” view of the femoral reamer when using the trans-portal technique for drilling the femoral tunnel, serving as the senior author’s preferred technique during ACL reconstruction. Unlike the standard notch view obtained with a 30° arthroscope from the anterolateral portal, this “top-down” view permits visualization of the far side (i.e., proximal aspect) of the ACL femoral tunnel aperture. The purpose of the study is to compare femoral tunnel length measurements using the “top-down” view with a 70° arthroscope to reference the far side of the femoral tunnel aperture versus measurements from the standard notch view using a 30° arthroscope to reference the near side. The authors hypothesized that femoral tunnel length measurements obtained from the “top-down” view would be more accurate than measurements obtained using the standard notch view.

## Methods

The study protocol was pre-approved by the author’s Institutional Review Board. Arthroscopic images of skeletally mature patients with closed physes undergoing primary ACL reconstruction by a single fellowship-trained Orthopaedic sports surgeon between July 1, 2014 and December 31, 2016 were retrospectively reviewed for study inclusion. As such, no formal patient consent was required. Skeletally immature patients and those with prior bony knee surgery were excluded. Demographic data including patient age, sex, height, weight and laterality of surgery were recorded.

### Surgical Technique

**Figure 1:**
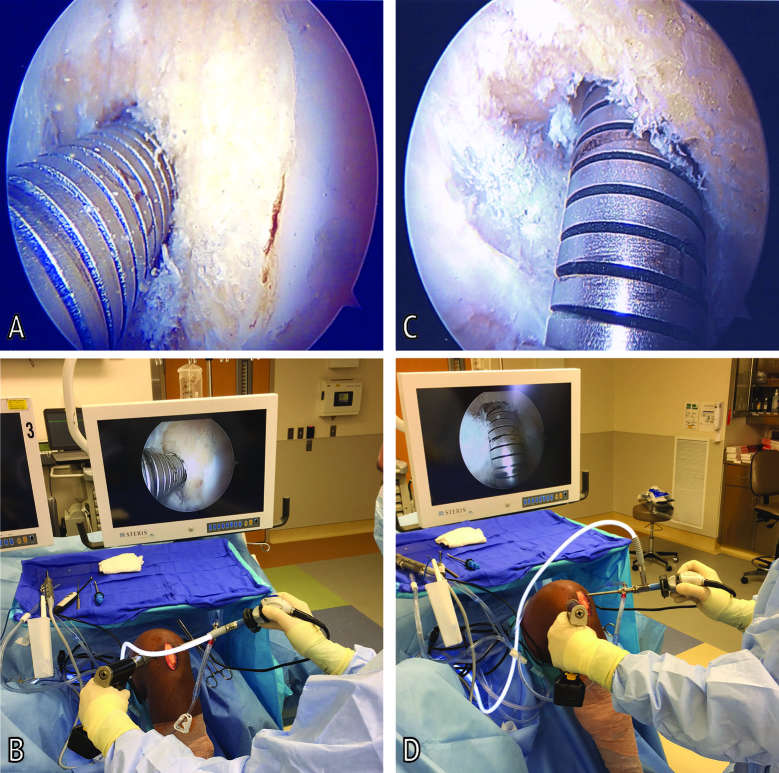
Arthroscopic images of a left knee during femoral tunnel reaming to obtain femoral tunnel reamer measurements. **(A)** Standard notch view **(B)** Surgeon hand and camera position for obtaining standard notch view **(C)** Top-down view **(D)** Surgeon hand and camera position for obtaining top-down view.

A standard 30° arthroscope was used for the diagnostic arthroscopy and to address any chondral or meniscal pathology. It is the senior author’s preference to use a 70° arthroscope for the ACL reconstruction portion of the operation to view the intercondylar notch and ACL footprint, perform tunnel drilling, and visualize graft insertion. At the time of femoral tunnel drilling using a flexible reamer (Clancy Flexible Reamer, Smith and Nephew, Memphis, TN) the 70° arthroscope is placed in the standard anterolateral portal looking laterally at the reamer. The surgeon stopped the reamer and obtained the first of two images of the reamer within the tunnel. Despite using a 70° arthroscope, the image obtained is identical to that obtained with a 30° arthroscope given then positioning of the light cord/camera lens. (Figure 1A,B) Care was taken to ensure a hash mark number label was clearly visible on the reamer in the image as reference for measurements of tunnel depth. The second image was obtained by rotating the camera 90° to achieve the “top-down” view, which orients the visual field looking down on the reamer to visualize the far side (i.e., back-side) of the reamer and femoral tunnel aperture. (Figure 1C,D) Care was again taken to ensure inclusion of a hash mark number label on the reamer for reference of tunnel depth.

### Measurements and Calculations

**Figure 2:**
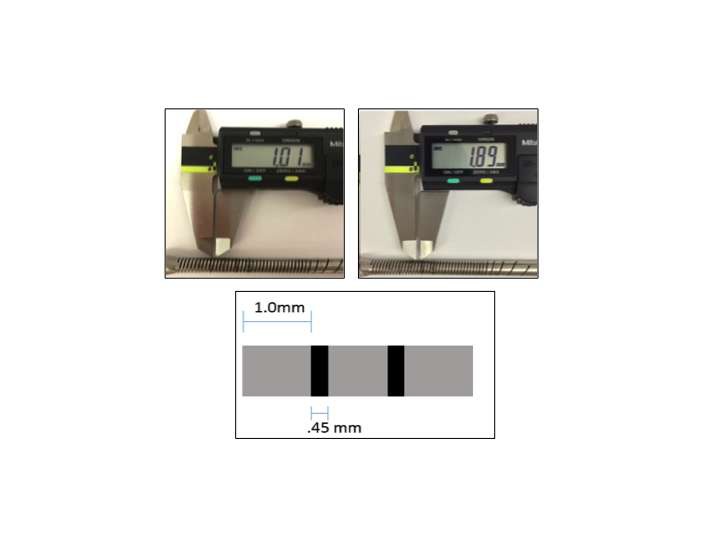
Digital caliper measurement of flexible reamer links and gaps for femoral tunnel length measurements.

The flexible reamer’s links between each 5 mm labeled hash mark were measured using a digital caliper with accuracy to 0.01 mm (Mitutoyo, Mitutoyo Corp., Model Japan). A single metal link was measured to be 1.01 mm long. (Figure 2A) The length of a single link and the two adjacent gaps on either side was measured as 1.89 mm long. (Figure 2B) These two measurements were rounded to the nearest 0.1 mm. Thus, the length of the space between two links was determined by subtracting the length of a single link (1.0 mm) and then dividing the remaining length by two, given the presence of two inter-link gaps. [i.e., (1.9–1.0 mm)/2 = 0.45 mm]. (Figure 2C) Thus, the femoral tunnel length could be accurately calculated from arthroscopic images by referencing the visualized hash mark number label and subtracting the link lengths (1.0 mm) and the inter-link gap lengths (0.45 mm) until the point at which the reamer intersected with the femoral tunnel wall aperture. The femoral tunnel length measurements were determined from the surgical arthroscopic images using the standard notch view and the “top-down” view for each patient.

### Statistical Analysis

An a priori power analysis was conduced to determine the minimum number of patients necessary to detect a significant difference in femoral tunnel length measurements between views. Using an initial set of 20 measurements (*n* = 10 patients), SD was assumed to be 0.63 mm. With an alpha-level of 0.05 and power set to 95%, the minimum number of patients needed to detect a 0.5 mm difference was calculated to be 54 patients.(16)

All data were analyzed for normality using the Shapiro-Wilk test. An intraclass correlation coefficient (ICC) for femoral tunnel length measurements was calculated by the senior author at two separate time points (minimum: 2 weeks apart) in all patients (*n* = 54 patients; *n* = 108 images) while an interclass class correlation coefficient was calculated in 26 patients (*n* = 52 images) between two authors. Following established recommendations, an ICC of <0.4 was determined to be poor, 0.4–0.75 to be fair to good, and >0.75 to be excellent.(17, 18) Paired samples *t-*test was used to test the null hypothesis that no significant difference would be present between measurements obtained via the standard notch view versus measurements obtained via the “top-down” view. An independent sample *t*-test was used to compare dichotomous variables (patient sex, laterality of surgery) in paired measurements obtained from the two views. Pearson’s correlations were used to compare age, height, and weight to the difference in paired measurements obtained from the two views. A *p*-value of <0.05 was used to determine statistical significance. All statistical analysis was performed using SPSS (Version 23, IBM, Armonk, New York) software.

## Results

A total of 60 patients underwent ACL reconstruction during the study period with imaging and documentation of both standard-notch and “top-down” views for femoral tunnel length measurement. Six patients were excluded due to bony debris obscuring the reamer’s hash mark number labels, preventing accurate tunnel length measurement in at least one of the views. Mean age of the 54 patients included for final analysis was 26 ± 9.8 years (range, 15 to 51 years) with a mean height and weight of 69 ± 4.4 inches (range, 58–77 inches) and 84.0 ± 18.7 kilograms (range, 54.0–136 kilograms), respectively. The study group included 19 females and 35 males. Twenty-seven patients underwent surgery to the left knee while 27 had surgery on the right knee.

**Figure 3:**
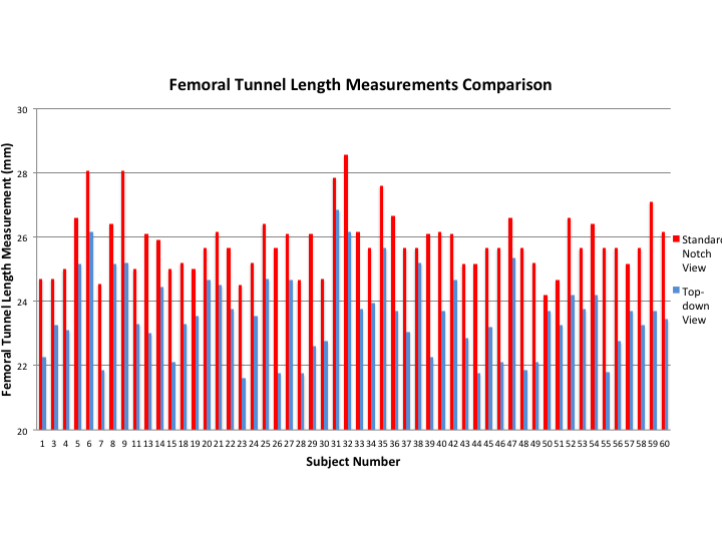
Comparison of femoral tunnel length measurements obtained by the standard notch view and the top-down view.

**Figure 4:**
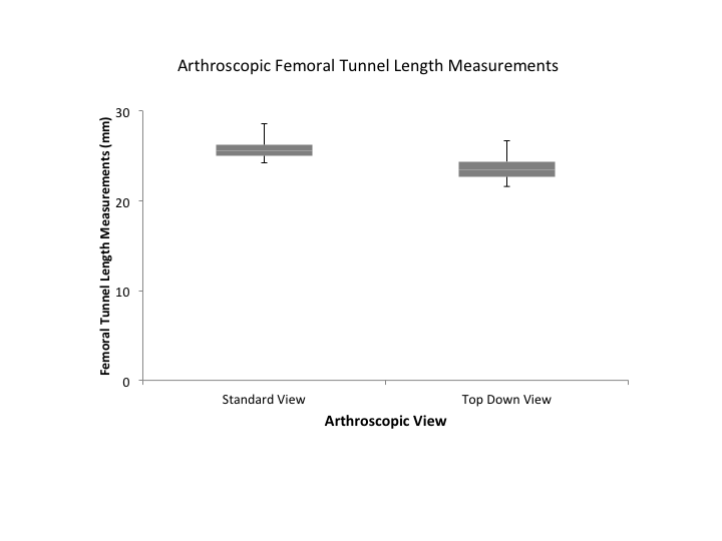
Box plots of femoral tunnel length measurements in millimeters by arthroscopic technique. Upper and lower hinges represent 25 and 75% quartiles; middle represent median or 50% quartile.

Measurements of both the standard notch view and “top down” view produced excellent intra-observer (0.0877, 0.932; respectively) and inter-observer (0.916, 0.960; respectively) values. Femoral tunnel length measurements obtained via the standard notch view were significantly longer than those obtained via the “top-down” view (*p* < 0.001; mean 2.24 ± 0.868 mm longer) (Figures 3 and 4). Univariate analysis showed that the difference in femoral tunnel length measurements obtained via the standard notch-view versus the top-down view did not vary significantly with age (r = 0.12, *p* = 0.40), height (r = 0.013, *p* = 0.92), or weight (r = 0.042, *p* = 0.76). There was no significant difference in mean femoral tunnel length measurements between females (mean, 2.27 ± 0.666 mm) and males (mean, 2.22 ± 0.974 mm) (*p* = 0.852). Furthermore, no significant difference in femoral tunnel length measurements was appreciated based on surgical laterality (mean, 2.08 ± 0.776 mm for left versus 2.40 ± 0.94457 mm for right; *p* = 0.179).

## Discussion

The purpose of this investigation was to better understand different in femoral tunnel length measurements during ACL reconstruction comparing the standard notch view using a 30° arthroscope versus the “top down” view with a 70° arthroscope. The authors found that in 54 subjects undergoing ACL reconstruction, measurements of femoral tunnel length via the standard notch view were significantly longer than those obtained using a “top down” view. Patient sex, age, height, weight and surgical laterality had no significant impact on mean femoral tunnel length measurements. Data from this investigation adds to the growing body of research regarding femoral tunnel aperture morphology and its clinical implications during ACL reconstruction.(13, 19, 20)

Multiple studies have implicated non-anatomic femoral tunnel placement as the reason for ACL reconstruction failure and instability post-operatively (21–23) as current efforts towards achieving optimal stability and outcomes have focused on anatomic ACL reconstruction techniques.(4, 24) Obtaining anatomic femoral tunnel placement and subsequent tunnel length measurement relies on adequate visualization of the native ACL footprint. Numerous techniques have been proposed for gaining better visualization of the lateral femoral condylar wall within the notch, including a modified mid-patellar portal (25) and a central accessory medial portal.(26) By using a 70° arthroscope through the standard anterolateral portal, the “top-down” view described in this study provides the surgeon with an unobstructed view of the far side of the femoral tunnel aperture without the need for extra portals. By providing a perpendicular view of the reamer at the femoral tunnel aperture, the authors found excellent intra- and inter-observer reliability values, indicative of the high reproducibility of measurements using the “top down” view. In their investigation utilizing a 3-dimensional knee model, Hoshino et al. further demonstrated the ability to reduce distortion and consequent inaccurate measurement readings using the 70° arthroscope when compared views obtained using 0° and 30° arthroscopes.(27)

Compared to the standard notch view, the “top down” view avoids over-estimation of femoral tunnel length measurements. This effectively mitigates potential complications associated with graft-tunnel length mismatch, which has been shown to be crucial in order to ensure optimal outcomes following ACL reconstruction.(28, 29) When using a bone-patellar tendon-bone graft or osseous grafts such as an Achilles or quadriceps tendon, over-estimation of the femoral tunnel length may lead to a proud bone plug within the notch and/or inadequate bone-plug interface at the tibial tunnel, compromising the integrity of interference screw fixation (30), leading to intra-operative or postoperative complications.(31) Arguably, a buffer distance may be built into graft length and tunnel drilling calculations. However, having a femoral bone plug that ends flush with the femoral tunnel aperture within the notch avoids potential complications associated with a recessed femoral bone plug such as the “windshield wiper effect,” thought to result in tunnel widening and graft abrasion.(32, 33)

Moreover, for a soft-tissue graft, overestimation of femoral tunnel length can lead to placement of an inadequate amount of the graft within the femoral tunnel.(34) Lee et al. demonstrated that such grafts incompletely fill femoral tunnel apertures and tend to rest off-center within the tunnel.(35) Accordingly, a discordant measurement of osseous tunnel length versus graft length may be amplified with the graft’s final eccentric resting position. Previous investigations have examined the temporal changes in the cross-sectional area of the femoral tunnel aperture, enlarging with time (36) with slower remodeling of the bone-tendon interface at the intra-articular aperture.(37) The consequences of these natural history changes in ACL reconstruction grafts are magnified with increased motion at the tunnel aperture, resulting in lower rates of healing at sites of motion.(38)

This study was not without limitations. A single surgeon obtained all arthroscopic photographs used to measure femoral tunnel length during surgery. While use of the 70° arthroscope is the senior author’s preference, inviting the potential for observer bias, the excellent inter-observer reliability values obtained demonstrate the validity of the methods utilized and results obtained. However, additional studies utilizing multiple different surgeons is warranted to further validate the conclusions and surgical technique of the “top down” view. Second, if suspensory fixation techniques with an adjustable loop fixation function are utilized, then concerns about graft-tunnel mismatch become less pertinent since the loop portion of the graft-button construct is adjustable following passage of the graft.(20) Lastly, post-operative functional outcome scores and complications rates were not analyzed, as such despite the reported significant difference in femoral tunnel length overestimation using the standard notch view, the clinical relevance of this finding cannot be extrapolated and is beyond the scope of this investigation.

## Conclusions

The “top-down” view using a 70° arthroscope provides a viable alternative to the standard notch view with a 30° arthroscope for visualizing the anatomic ACL footprint. The “top down” view helps avoid tunnel length overestimation by referencing the backside of the reamer for tunnel length measurements, especially in cases where lateral femoral intercondylar notch wall obliquity is high or the tunnel drilling obliquity is high. By providing a more accurate assessment of femoral tunnel length, the surgeon can plan appropriately for final tunnel length preparation and graft length preparation, facilitating more advantageous ACL reconstruction by avoiding complications associated with graft-tunnel length mismatch. Future long-term prospective studies examining functional outcomes and complication rates following ACL reconstruction utilizing the “top-down” view with a 70° arthroscope versus the standard notch view with a 30° arthroscope are necessary to better understand the clinical impact of technique on outcomes following reconstruction.

## Ethics

This study was carried out in accordance with the recommendations of the University Hospitals Cleveland Institutional Review Board. The protocol was approved by the University Hospitals Institutional Review Board who deemed that written consent from patients was not required due to the retrospective nature of the investigation.

## Author Contributions

SMJ: project conception and design, measurements, data analysis, manuscript preparation. MRK: project conception and design, measurements, data analysis. DMK: data analysis and manuscript preparation. JEV: project conception and design, obtained surgical images, manuscript preparation.

## Conflict of Interest Statement

The authors declare that the research was conducted in the absence of any commercial or financial relationships that could be construed as a potential conflict of interest.
